# Purification and functional characterization of protoplasts and intact vacuoles from grape cells

**DOI:** 10.1186/1756-0500-3-19

**Published:** 2010-01-22

**Authors:** Natacha Fontes, Rui Silva, Céline Vignault, Fatma Lecourieux, Hernâni Gerós, Serge Delrot

**Affiliations:** 1 Centro de Investigação e de Tecnologias Agro-Ambientais e Biológicas (CITAB). Portugal; 2 Departamento de Biologia, Universidade do Minho, Campus de Gualtar, 4710-057 Braga, Portugal; 3 Centro de Biologia Molecular e Ambiental, Departamento de Biologia, Universidade do Minho, Braga, Portugal; 4 Laboratoire de Physiologie Moléculaire du Transport des Sucres chez les Végétaux, Université de Poitiers, Poitiers, France; 5 UMR 1287 Ecophysiology and Grape Functional Genomics, University of Bordeaux, INRA, Institut des Sciences de la Vigne et du Vin, Domaine de la Grande Ferrade, 210 chemin de Leysotte, 33883 Villenave d'Ornon, France

## Abstract

**Background:**

During grape berry ripening, the vacuoles accumulate water, sugars and secondary metabolites, causing great impact in plant productivity and wine quality. However, the molecular basis of these compartmentation processes is still poorly understood. As in many species, the major bottleneck to study these aspects in grapevine is to obtain highly purified vacuoles with a good yield. The present paper describes an isolation method of protoplasts and intact vacuoles from grape berry cells and their functional characterization by transport and cytometric assays.

**Findings:**

Protoplasts were prepared by enzymatic digestion of grape cells, and vacuoles were released and purified by a Ficoll step gradient centrifugation. The tonoplast stained strongly with the fluorescent dye FM1-43 and most vacuoles maintained an internal acidic pH, as assessed by Neutral Red. Flow cytometry analysis of vacuole samples incubated with the calcium-sensitive fluorescent probe Fluo-4 AM revealed a well-defined sub-population of intact vacuoles. As assessed by the pH-sensitive probe ACMA, intact vacuoles generated and maintained a pH gradient through the activity of V-ATPase and V-PPase and were able to transport Ca^2+ ^via a proton-dependent transport system.

**Conclusions:**

Highly pure, intact and functional protoplast and vacuole populations from grape cells were obtained with the present method, which revealed to be fast and efficient. The capacity of the vacuole population to sequester protons and accumulate Ca^2+ ^strongly suggests the intactness and physiological integrity of these extremely fragile organelles. Grapevine protoplasts and vacuoles may be used as models for both basic research and biotechnological approaches, such as proteomics, solute uptake and compartmentation, toxicological assessments and breeding programs.

## Findings

### Enzymatic digestion of grape cells yields highly pure, viable and homogeneous populations of protoplasts

Protoplasts were prepared from *Vitis vinifera *L. cells (CSB, Cabernet Sauvignon Berry). Cells were cultivated in liquid mineral medium supplemented with 2% (w/v) sucrose. The method of Greuter and Keller [1] to isolate protoplast from *Stachys sieboldii *tubers was adapted for grape cells and optimized by introducing several changes, including the composition of the media, enzyme proportion and purification steps. Protoplasting was performed by enzymatic digestion of the cell walls (450 × 10^6 ^cells) with 0.007% (w/v) cellulase Y-C and 0.0007% (w/v) pectolyase Y-23 (Kyowa chemical products CO., LTD) in a final volume of 50 ml. Digestion occurred in Gamborg B5 Medium supplemented with 0.4 M sucrose, under shaking (50 rpm), at pH 5.8 and 22°C. Different digestion periods of 4 to 12 h were tested. The resulting protoplasts were gently collected and subsequently purified. Initially, protoplasts were separated by floating, at 150 × *g *for 8 min, and subsequently washed with the same medium. A discontinuous gradient was prepared by overlaying 1 volume of a solution containing 0.05 M glucose, 154 mM NaCl, 125 mM CaCl_2 _and 5 mM KCl, pH 5.8, on the protoplast suspension, followed by centrifugation at 150 × *g *for 8 min. Protoplasts were recovered from the interface of the gradient, resuspended in 3 volumes of the glucose-containing medium and sedimented for 8 min at 150 × *g*. The pellet was washed in 0.4 M mannitol, 15 mM MgCl_2_, 5 mM Mes, pH 5.8, resuspended in the same medium and stored at 4°C. Protoplasts were counted in a Malassez chamber under the light microscope. A protoplast yield of 13% was obtained as a result of a 12 h digestion protocol, decreasing approximately to 6% when the digestion lasted 4 h.

**Figure 1:**
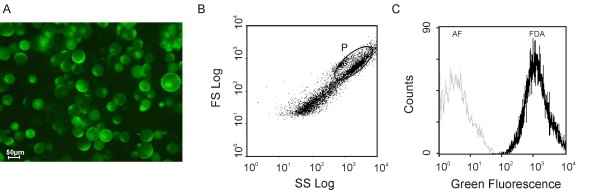
**Microscopical and flow cytometric analysis of a protoplast population purified from grape cells**. Protoplasts were labelled with FDA and observed under UV light (epifluorescence, A). Flow cytometric analysis of the protoplast population labelled with FDA: scattergram for grape cells protoplasts after FDA staining (B) and overlay of green fluorescence and autofluorescence histograms of the same protoplast suspension of gated region P (C).

The viability of the protoplasts was tested with the fluorescent dye fluorescein diacetate (FDA). The intact plasma membrane is permeable to FDA, and FDA is converted into a green fluorescent dye, fluorescein, by internal esterases, displaying a green fluorescence in viable cells [2]. Figure 1 depicts a typical protoplast population labelled with FDA observed under UV light (epifluorescence, A). Comparison of the epifluorescence light with the visible light images (results not shown) showed that most protoplasts remained viable immediately after isolation, displaying an intense green fluorescence, regardless of the duration of the digestion step (4 or 12 h). However, when a 4 h-digestion protocol was used, the viability was always higher, reaching 100% in some cases. The same conclusions were achieved after flow cytometry analysis, as described below.

Flow cytometry analysis requires that microscopical biological particles be in suspension. It allows the simultaneous quantification of multiple fluorescence emissions in the same cell or biological particle, and scattered light related to morphology [3]. Therefore, individual cells or sub-cellular particles from heterogeneous subpopulations can be physically isolated on the basis of their fluorescence or light scatter properties [4]. In the present work, flow cytometry has been exploited in order to characterize the protoplast and vacuole samples and to individualize the subpopulations, allowing conclusions about the purity of each fraction. Flow cytometric analysis was performed in an Epics^® ^XLTM (Beckman Coulter) flow cytometer equipped with an argon-ion laser emitting a 488 nm beam at 15 mW. Green fluorescence was collected through a 488 nm blocking filter, a 550 nm long-pass dichroic and a 525 nm band-pass filter. For each sample, 20,000 protoplasts and 20,000 vacuoles were analysed at low flow rate. An acquisition protocol was defined to measure forward scatter (FS), side scatter (SS) and green fluorescence (FL1) on a four decades logarithmic scale. Data were analysed by WinMDI 2.8 software. The analysis of the biparametric histograms, plotting log SS against log FS, revealed some heterogeneity in both relative complexity and size of the protoplast population (Figure 1B). However, the subpopulation of protoplasts can be easily identified, since it easily stains well with FDA (gated region P). Above and to the right of this region, there is also a subpopulation that probably consists of protoplast aggregates. The subpopulations with the lowest scatter (below and to the left of region P) correspond mainly to submicroscopic particles - as some cell debris and cell wall residues - of relative low complexity and size, which were co-purified with the protoplasts. Figure 1C depicts the overlay of the green fluorescence and auto-fluorescence histograms of the gated region P. Quantification of the percentage of FDA positive stained cells indicates that the protoplasts population exhibits a high percentage of viability. These findings were previously summarized in the book chapter by Papadakis et al. [5].

### The lysis of grape protoplasts yields highly pure and intact vacuoles

Vacuoles were released upon the protoplast osmotic lysis at a relatively high temperature and were purified by a Ficoll step gradient centrifugation. The methodology was adapted from the protocol used to obtain vacuoles from Arabidopsis protoplasts [6]. The protoplast suspension was added to 2.5 volumes of the pre-warmed (37-45°C) lysis buffer, a solution with reduced osmotic strength containing 0.2 M mannitol, 10% Ficoll (w/v), 15 mM EDTA, 10 mM MOPS, pH 8.0, supplemented with 0.1% BSA and 2 mM DTT, resulting in the release of intact vacuoles. The vacuoles were collected from the vacuole buffer layer after a one-step Ficoll gradient centrifugation of 15 min at 1000 × *g*. The discontinuous gradient was optimized as follows: one layer of the lysis mixture (10% Ficoll, w/v), one layer of 3.0% Ficoll (w/v) and one layer of vacuole buffer containing 0.5 M mannitol, 10 mM MOPS, pH 7.5 and a protease inhibitor cocktail (*Complete*, Roche Applied Science, Germany), in the proportion of 7:3:1 volumes. The 3.0% Ficoll solution was prepared by diluting the lysis buffer with vacuole buffer. The vacuoles were counted on a Malassez chamber under the light microscope. In a typical fractionation procedure, an average amount of 4.0 × 10^6 ^vacuoles was obtained, corresponding to about 12 % of the total number of protoplasts (purified by a 12-h digestion protocol) subjected to lysis. Since the yield of intact vacuoles was always higher when protoplasts were isolated by a 12-h digestion protocol (not shown), this digestion duration was used in all subsequent experiments. Cytosolic glucose-6-phosphatase was used as a marker enzyme to monitor vacuole purification [7]. The specific activities of the protoplast preparation and vacuole preparation were 715 and 14.6 (nmol min^-1 ^mg prot^-1^), respectively. Only 2% of the marker enzyme was recovered in the vacuolar fraction, indicating that this sample was strongly depleted in protoplasts and cytosolic contaminations. This conclusion was further supported by microscopic observation and flow cytometry analysis.

**Figure 2:**
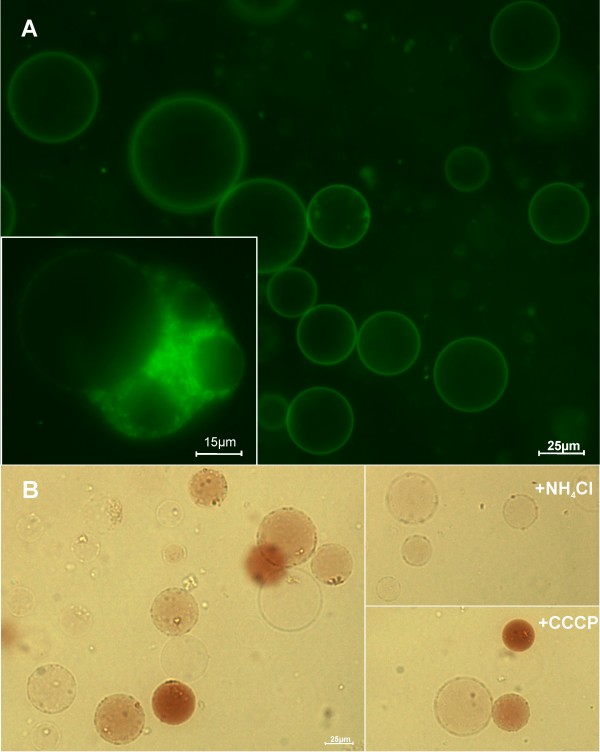
**Microscopic characterization of intact vacuoles purified from grape protoplasts**. Intact vacuoles, purified after protoplast lysis, labelled with the fluorescent membrane marker FM1-43 (A; *inset*: membranous bodies attached to the central vacuole forming an "impure vacuole") observed under the fluorescence microscope. Intact vacuoles labelled with neutral red in the absence and in the presence of 2.5 mM NH_4_Cl and 25 μM CCCP (B).

To visualize the vacuoles with the fluorescence microscope, the styryl dye FM 1-43 was used as described earlier [8]. This fluorescent probe exhibits weak fluorescence in aqueous medium, but shines brightly when inserted into membranes [9]. Incubation resulted in a strong staining of the tonoplast (Figure 2A), demonstrating the intactness of these extremely fragile organelles. Vacuole preparations do not contain just "naked" vacuoles, and some vacuoles seem to be contaminated with cytoplasmic remnants. Such "impure vacuoles" were easily identified under the light or fluorescence microscope after FDA labelling, as reported before [10], as well as by incubation with the styryl dye FM 1-43 (Figure 2A, *inset*). This figure suggests that several structures belonging to the "vacuolar apparatus" (i.e., vacuoles and those membranous bodies that are either committed to become vacuolar or that have immediately completed a vacuolar function) remained attached to the central vacuole after protoplast lysis. The virtual absence of intact protoplasts in the vacuole preparations was confirmed by the lack of labelling by FDA that only stains the cytoplasm.

Most of the intact vacuoles, ranging in size from 10 to 50 μm, maintained an internal acidic pH and exhibited a red colour after being labelled with Neutral Red, a lipophilic phenazine dye (Sigma-Aldrich), in spite of their resuspension in a buffer at pH 7.5 (Figure 2B). This acidity was relatively stable and was not completely abolished by the incubation with 100 μM CCCP. This may be due to the buffering capacity of organic acids accumulated in the vacuoles, but we must not discard the fact that higher protonophore concentrations could promote red colour dissipation. However, 2.5 mM NH_4_Cl almost completely abolished the pH gradient across the majority of the vacuoles.

**Figure 3:**
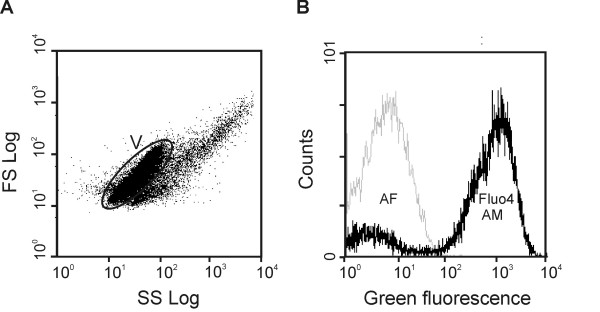
**Flow cytometric analysis of a vacuole population purified from grape protoplasts**. Scattergram (dot plot) of a vacuole suspension after Fluo-4 AM staining (A) and overlay of green fluorescence histograms of stained and autofluorescence of vacuole suspension for gated region V (B).

The flow cytometry analysis of vacuole samples is depicted in Figure 3A and 3B. The scattergram shows a well-defined subpopulation in the centre of the histogram, corresponding to the vacuole population (gated region V). After an incubation period of 15 min at room temperature with the calcium probe Fluo-4 AM (Molecular Probes, Eugene, OR, USA), the fluorescence of the gated region V increased (Figure 3B), indicating the vacuole ability to accumulate calcium. The use of flow cytometry to analyze the functional properties of isolated subcellular particles is less frequent than its application in whole cell studies. However, most current instruments are sensitive enough for subcellular analyses. In the present work, flow cytometric analysis allowed us to identify well-defined and viable subpopulations of protoplasts and vacuoles, based on viability (FDA) and calcium content (Fluo-4 AM), respectively (Figure 1 and 3). These findings regarding vacuole characterization by microscopy and flow cytometry analysis were previously summarized in the book chapter by Papadakis et al. [5]. So far, only spinach chloroplasts [11] and rice protoplasts [12] have been studied by flow cytometry. The present results open new perspectives for future work concerning the study of calcium transport across the tonoplast with calcium sensitive fluorescent probes and flow cytometry analysis.

### Intact vacuoles from grape cells are physiologically active organelles

**Figure 4:**
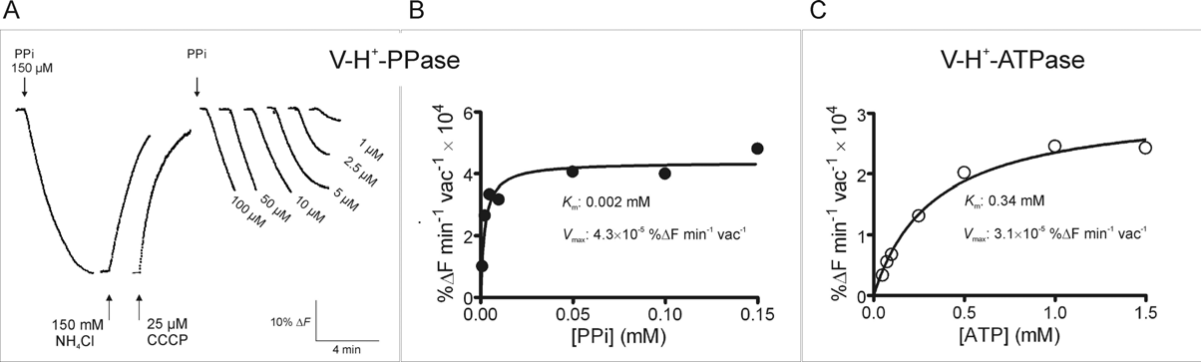
**Proton pumping activity in intact vacuoles purified from grape protoplasts**. The accumulation of protons was determined by measuring the fluorescence quenching of ACMA [14,15] in 2 × 10^5 ^vacuoles. Typical fluorescence signals of the initial velocities of proton pumping by V-H^+^-PPase after addition of 0.1 to 150 μM PPi (A) and the corresponding Michaelis-Menten plot (B). Michaelis-Menten plot of the initial velocities of proton pumping by V-H^+^-ATPase as a function of ATP concentration (0.05 to 1.5 mM) (C). The results presented in B and C show the average values of two independent experiments.

Monitoring the transmembrane proton gradient in intact vacuoles is a proper approach to study the mechanisms of vacuolar acidification, because intact vacuoles are physiologically closer to the *in vivo *plant system than tonoplast vesicles. The low pH of the vacuole of fruit cells is the result of two processes: 1) pumping protons across the tonoplast, which directly results in a drop of vacuolar pH, and 2) synthesis and accumulation of organic acids in the vacuolar sap [13]. For proton-pumping measurements, intact vacuoles were labelled with ACMA (9-amino-6-chloro-2-methoxyacridine), a highly sensitive pH-dependent fluorescent dye. The fluorescence quenching of ACMA was measured using a Perkin-Elmer LS-50 [14,15]. Vacuoles (between 1.0 × 10^4 ^and 1 × 10^5^) were added to the assay cuvette (2.0 ml), containing 100 mM KCl, 2 mM MgCl_2_, 0.1% BSA (w/v), 10 mM MOPS-Tris pH 7.2 and 2 μM ACMA. The optimal concentration of Mg^2+ ^in the assay medium was previously adjusted taking into account that the cation forms insoluble complexes with PP_i_, and then changing PP_i_/Mg^2+ ^ratio modifies the H^+ ^pumping activity of V-PPase (Maeshima, M., personal communication). Figure 4 shows the PPi-dependent and ATP-dependent H^+ ^pumping activities across the membrane of intact vacuoles from grape cells, as measured by the fluorescence quenching of ACMA. Both NH_4_Cl and CCCP induced a prompt recovery of ACMA fluorescence, demonstrating the generation of a pH gradient. In this biological system, the V-PPase seems to be the main tonoplast proton pump, generating a pH gradient 1.4-fold greater than the V-ATPase, counting with 170 times less substrate concentration: the *V*_max _value for the V-PPase proton pumping was 4.3 × 10^-5 ^%ΔF min^-1 ^vac^-1^, while the *V*_max _for V-ATPase was 3.1 × 10^-5 ^%ΔF min^-1 ^vac^-1^. Indeed, the *K*_m _of V-PPase proton pumping was determined to be 2.0 μM PPi, while the *K*_m _for the V-ATPase was 340 μM ATP.

**Figure 5:**
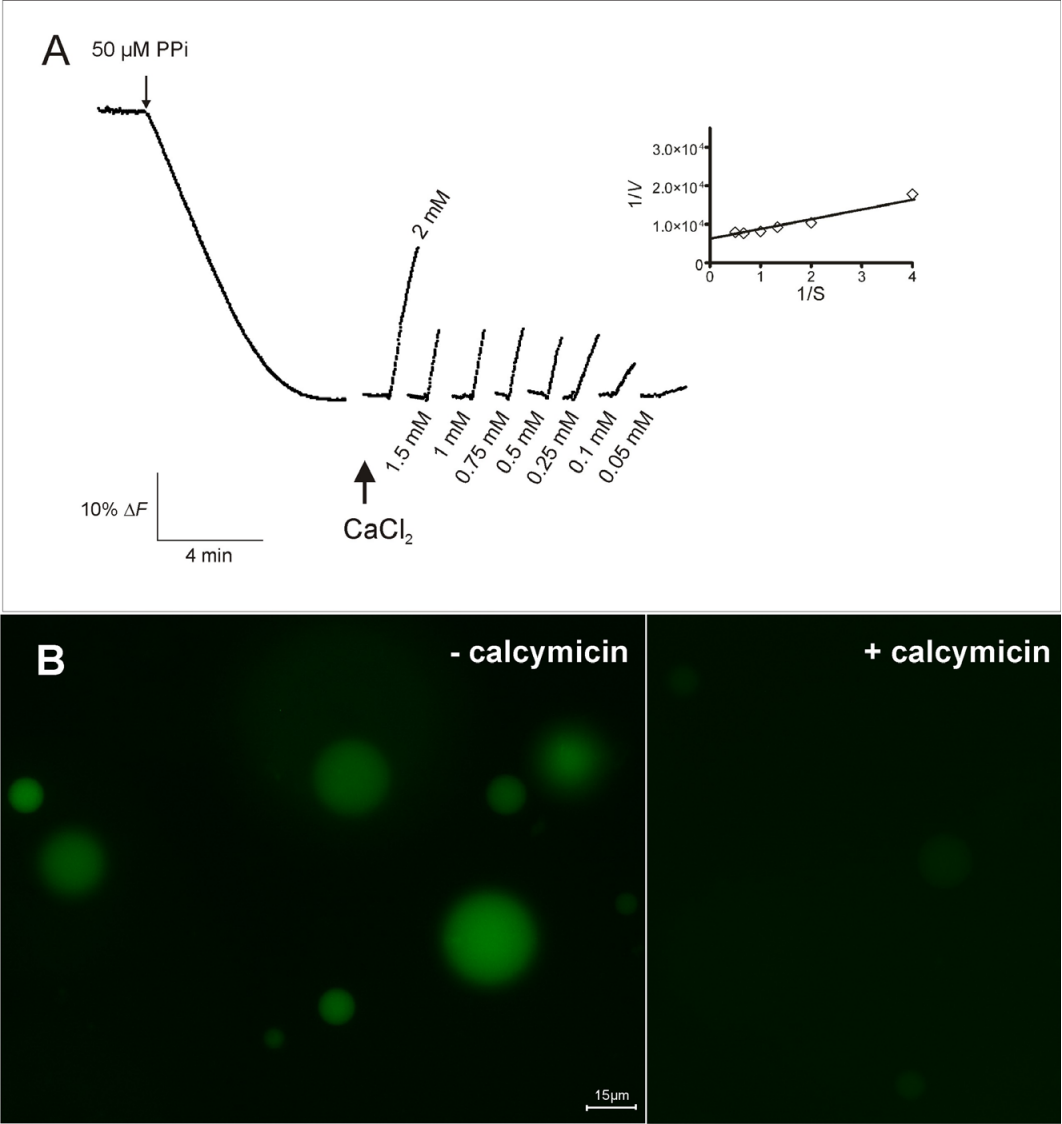
**Study of the involvement of H^+^-dependent Ca^2 ^uptake in intact vacuoles purified from grape protoplasts. **Effect of Ca^2+ ^on the pre-formed transmembrane proton gradient (A). *Inset*: Lineweaver-burk plot of the initial velocities of fluorescence recovery as function of Ca^2+ ^concentration. The fluorescence signals shown are representative of at least three independent experiments. Intact vacuoles labelled with the calcium fluorescent probe Fluo-4 AM in the absence and in the presence of 300 μM calcymicin, observed under the fluorescence microscope (B).

The ability of CaCl_2 _to dissipate a pre-established ∆ pH gradient across the tonoplast was used to assess the involvement of a Ca^2+^/H^+ ^antiport system. The addition of CaCl_2 _to energized intact vacuoles through the activation of V-PPase resulted in an immediate dissipation of the proton gradient (Figure 5A). Initial velocities of fluorescence recovery after Ca^2+ ^addition followed a Michaelis-Menten kinetics, and a *K*_m_= 0.4 mM and a *V*_max_= 1.68 × 10^-4 ^%ΔF min^-1 ^vac^-1 ^were estimated (Figure 5A, *inset*). A vacuole sample (100 μl) was also labelled with 0.5 μM of the calcium probe Fluo-4 AM, for 15 min at room temperature, prior to observation under the fluorescent microscope. The high fluorescence observed was substantially decreased after the addition of 300 μM calcymicin (Figure 5B). These data, together with those from flow cytometry analysis, suggest that a functional Ca^2+^/H^+ ^antiporter is working in the purified vacuoles from grape cells, which is likely to contribute to the accumulation of Ca^2+^.

Above results show that two distinct primary proton pumps, the vacuolar ATPase and the vacuolar inorganic pyrophosphatase (V-PPase), generate a proton electromotive force, which, in turn, allow the secondary active transport of several compounds that are accumulated in the vacuole, as it has been shown for Ca^2+ ^uptake. However, we must not ignore that the long digestion period used to purify the protoplasts can affect both the vacuolar contents and the activity of the transporter proteins. The predominant activity of the V-PPase compared to that of V-ATPase in intact vacuoles (Figure 4) is in agreement with the earlier results [14,16-18]. The capacity of the vacuole population to sequester protons strongly suggests the intactness and integrity of these extremely fragile organelles.

## Conclusions

Structural studies [19-21] and, more recently, proteomic analysis [22-26] have elucidated some aspects of vacuole function and biogenesis, and have generated an unpredicted interest in the purification of this extremely fragile organelle. During the ripening of fleshy fruits, the vacuoles accumulate water, sugars and secondary metabolites [13,27,28]. In spite of its importance for crop yield and quality, the molecular basis of these compartmentation processes is still poorly understood for grapevine. As in many species, the major bottleneck to study these aspects in grapevine is to obtain highly purified vacuoles with a good yield. This work describes the preparation of intact and viable vacuoles from grape cells suspensions, so as to demonstrate their feasibility as a model system to study the mechanisms underlying vacuolar compartmentation. A fast and efficient method has been developed to isolate highly pure and intact protoplast and vacuoles from grape suspension-cultured cells. Protoplasts and vacuoles may be used as models for both basic research and biotechnological approaches, such as proteomics, solute uptake and compartmentation, toxicological assessments and grapevine breeding programs.

## Competing interests

The authors declare that they have no competing interests.

## Authors' contributions

NF carried out the experiments reported in the main manuscript, performed data processing and statistical analysis and participated in amending the draft. RS assisted flow cytometric analysis. FL and CV contributed to the optimization of the protoplast purification procedure. HG and SD conceived the study and wrote the manuscript. All the authors approved the final version.
